# A longitudinal analysis of the clinical practice of ketamine assisted psychotherapy with adolescents and young adults

**DOI:** 10.3389/fpsyt.2026.1886833

**Published:** 2026-09-21

**Authors:** Phil Wolfson, Cody Carroll

**Affiliations:** 1 The Ketamine Research Foundation, San Anselmo, CA, United States; 2 University of San Francisco, San Francisco, CA, United States

**Keywords:** adolescent mental health crisis, adolescent psychiatry, bipolar disorder, eating disorders, ketamine, ketamine assisted psychotherapy, major depressive disorder, psychedelic psychotherapy

## Abstract

**Introduction:**

Ketamine Assisted Psychotherapy (KAP) is a developing modality with broad impact on humans suffering with a variety of emotional and relationship difficulties that encompass many diagnostic entities. Our application of KAP has extended to and included the treatment of adolescents and young adults in a family therapy format. Given the increasing scope and complexity of the adolescent mental health crisis and the frequent failures to respond to conventional treatment, the promise of KAP deserves thorough investigation.

**Methods:**

We previously described the process of KAP in our adolescent population that included four case reports exemplifying our approach. Here we report on the longitudinal clinical experience of 19 adolescents and young adults (ages 14–24; mean age 19.3 ± 3.1 years; 11 female, 7 male, 1 non-binary) who completed three or more KAP sessions at a single community outpatient clinic.

**Results:**

BDI-II scores declined by an estimated 0.65 points per visit (95% CI: -0.90, -0.40) and HAM-A scores by 0.21 points per visit (95% CI: -0.44, +0.01), based on linear mixed-effects models. Approximately 63.2% of patients (12/19) achieved medium or large individual treatment effects on BDI; 47.4% (9/19) on HAM-A. Physician and patient reports indicated overall benefit in 68.4% of cases (13/19), including marked reductions in suicidality in at-risk patients. Ketamine was well tolerated across 167 documented sessions; nausea was the only notable adverse event, occurring in 5.4% of sessions and largely manageable with standard antiemetics. No hospitalizations, substance misuse, or evidence of neurological harm occurred over a median individual follow-up of 5.6 months (mean 10.1, range 0.9-36.4 months).

**Discussion:**

These findings suggest that KAP is feasible and well tolerated in a diagnostically complex adolescent population, with clinically meaningful reductions in depression and a similar trend for anxiety associated with continued treatment. To our knowledge, this is the first report to provide longitudinal quantitative data for office-based KAP in adolescents and young adults, and it establishes a foundation for and supports the design of future controlled trials in this population.

## Introduction

An alarming crisis in adolescent mental health is unfolding worldwide. Cross-national surveillance documents increase of up to 164% for anxiety and 119% for depression in recent years, with rates of severe psychological distress rising as high as 242% in some populations (2). In the United States, nearly 40% of high school students reported persistent sadness or hopelessness in 2023, 18% had experienced major depression, and 10% had attempted suicide. The adolescent suicide rate increased by 85.3% between 2007 and 2017, and drug overdose deaths among 15–19-year-olds surged during the COVID-19 pandemic, largely driven by fentanyl. These trends are disproportionately concentrated among LGBTQ+ youth, rural and American Indian/Alaska Native youth, and those with histories of poverty, foster care, or juvenile justice involvement (3).

Mood disorders in adolescents are increasingly common, often co-occurring across multiple diagnostic categories (4), and are associated with poorer outcomes than those with later onset (5), elevated suicide risk (6), and substantial long-term disability (7).

Longitudinal studies demonstrate significant associations between childhood mental disorders and children’s health as well as with their caregivers. Prospective and long-term studies of sub-threshold symptomatic adolescents and diagnosed adolescents indicate significant progression into adulthood of mental disorders, emphasizing the need for treatment, access to care, and the impact of social and economic factors (8–12). Young people are prescribed a multiplicity of medications for mental health concerns (13), and yet, many do not achieve remission, or do not respond to the current evidence-based or FDA-approved treatments (14).

Ketamine has been shown to yield rapid and clinically significant positive effects in adult treatment resistant mood disorders (15–18). Given this success of ketamine in adults, and the obvious need for effective treatment for adolescents we report on ketamine’s application as an assisted psychotherapy to adolescent mood disorders. A vital outcome of KAP is the successful reduction in suicidal ideation (19, 20). Given the new entry of ketamine to the treatment of adolescents and young adults, there is a very limited literature. We provide a review in order to situate our clinical report in this emerging hopeful treatment for adolescents.

Across the small but growing pediatric literature, ketamine and esketamine have shown a consistent signal of rapid antidepressant and anti-suicidal effect. Controlled trials report benefit within 24 hours to two weeks: intravenous ketamine versus midazolam in adolescent treatment-resistant depression (25) and repeated intravenous esketamine with reduced suicidal ideation (21), alongside open-label and single-dose studies in mixed diagnostic samples (24) and preliminary reports in autism spectrum disorder (26, 27). Systematic reviews and meta-analyses converge on the same conclusions: the effect is real and reasonably well tolerated, and higher doses may outperform the standard 0.5 mg/kg, but response is incomplete and heterogeneous, samples are small, follow-up is short and the predictors and neural correlates of response are only beginning to be identified (22, 23, 28–31). Two features of this evidence base motivate the present study. First, nearly all of it concerns intravenous, single- or few-dose administration in medical settings, with little attention to the subjective experience or to psychotherapy (32). Second, no published series describes office-based, psychotherapy-embedded ketamine (KAP) delivered longitudinally in adolescents and young adults. This is the gap the present report addresses.

The DiVincenzo meta-analysis of adults and adolescents (21) reports the safety and tolerability of ketamine for adolescents and the improved success of higher doses than the standard 0.5mg/kg with no substantial adverse effects, nausea being the most common and similar in frequency to adults. They cite a “rapid and robust antidepressant response in adolescents” with “better antidepressant outcomes for individuals who received longer treatment courses and higher doses” this applying both to adolescents and adults. Noteworthy is that the review appears to have come down to the same number of adolescents, 33, as in the Kim paper cited above (23). We address ketamine’s safety profile, including its use in pediatric and anesthetic contexts, in the Safety of Ketamine section below.

There are many papers on the intravenous use of ketamine for treatment without psychotherapy, generally in medical settings administered by anesthesiologists. See McInnes et al. for a thorough review (32). In this context attendance to the subjective experience of the ketamine patient is generally absent. Receiving a substance that causes small to large dissociative (psychedelic) experiences without having the ability to process these experiences tends to diminish their value and often leads to confusion and a sense of “where did I go?” and “what just happened to me?” (16, 17).

In contrast, the value of ketamine embedded in a psychotherapy context is now increasingly established in clinical practice and in the literature (16, 17, 33–35). Psychotherapy provides the support for a full expression of the subjective experience of ketamine within a conducive non-medicalized setting (17, 33–35) facilitating emotional stabilization, self-awareness, reconstitution and new and positive behavior. This is particularly relevant to the adolescent population, as most large studies have suggested that the combination of psychotherapy and medication are superior to either treatment on their own for anxiety and depressive disorders. A study of parent perspectives on ketamine suggested that although much of the research to date has been on IV ketamine, parents were desirous of having less invasive modes of administration. As a result, the authors speculated on the potential for ketamine assisted psychotherapy to help address some of the understandable parental concerns (36). Treatment of adolescents with KAP is at its core family therapy centered--full attention to the context of family life and parental support for this treatment are essential.

Sathappan and Yudkoff (37) report on the process of bringing parents into the ketamine treatment experience with their adolescent children. Informed consent for an adolescent requires not only legal consent from parents but also voluntary assent from the minor, balancing respect for an adolescent’s growing autonomy and their role in the familial and legal structure (38, 39). Ultimately, informed consent is a safeguard against harm, ensuring patients and families are treated with dignity and autonomy in medical settings. Wolfson et al. (1) previously reported extensively on the nature of KAP treatment for adolescents and young adults-citing positive outcomes with four patients with complex histories and presentations.

Throughout this report, our focus is on associations and descriptive trajectories, which may suggest but do not establish causal efficacy; the absence of a control group precludes attribution of observed changes to KAP specifically. The present study pursues the following objectives:

1. Describe the demographics and historical diagnostic complexity of this adolescent KAP clinical group
2. Characterize longitudinal change in the validated outcome measures we use
3. Evaluate the impact of psychedelic experiences
4. Review changes in specific risk presentations, including eating disorders and suicidality
5. Delineate the association between treatment duration and symptom improvement
6. Characterize the importance and impacts of at-home treatment and family therapy
7. Generate hypotheses for future clinical trials.

The organization of this paper is as follows. We first review the basic pharmacology of ketamine as relevant to our dosing context, then address its safety profile with particular attention to pediatric and long-term use. We next describe the KAP treatment approach and protocol before turning to our methods, including clinical group eligibility, outcome measures, and statistical approach. We then present our findings and close with a discussion of their clinical implications, limitations, and directions for future work.

### The basic pharmacology of ketamine

Ketamine presents in two enantiomers: the S(+) and the R(–) configurations and as a hydrochloride salt. Historically and in general use for off-label psychiatric indications, as well as for anesthesia and analgesia, is an equimolar racemic mixture of the two enantiomers. Although racemic ketamine has the broadest worldwide use, S(+)-ketamine came to market as a commercial patented nasal preparation for psychiatric use in 2019. In terms of potency the S isomer is four times more powerful than the R isomer. In the RS mixture that is in general use, the S isomer at 50% of the content has 75% of the effect. Both isomers when standardized for dose have the same effects. The half-life of ketamine is about 2.2 hours and there is one active metabolite, norketamine, with a half-life of about 1.2 hours (17).

Ketamine’s most probable mechanism of action is as an N-methyl-D-aspartic acid (NMDA) glutamate receptor antagonist. It may well be that its principal site of action is at a specific NMDA receptor that has a dual capacity as a locus for both antidepressant and dissociative attributes. When NMDA receptors on gamma-aminobutyric acid (GABA)-ergic neurons are antagonized, downstream glutamatergic neurons are disinhibited. This increased glutamatergic activity impacts neural signaling, synaptic plasticity, and connectivity. It is posited based on animal models that ketamine-induced synaptic potentiation and proliferation may play a key role in eliciting antidepressant effects. Ketamine also impacts other neurotransmitter systems, affecting cholinergic, opioidergic, monoaminergic, and GABAergic functions [see Wallach and Brandt for a comprehensive review (40)].

Beyond acute NMDA-receptor antagonism, converging preclinical evidence indicates that ketamine’s more durable effects are mediated by a burst of activity-dependent synaptic plasticity: the glutamate surge following GABAergic disinhibition activates AMPA receptors and downstream BDNF-TrkB and mTORC1 signaling, driving rapid synaptogenesis in prefrontal circuits (41–43). This has given rise to the concept of a post-dose “plasticity window,” a transient period of heightened neural malleability in the hours to days after administration, during which new learning and, in the KAP model, psychotherapeutic integration may be especially consequential; recent work extends this idea to the reopening of critical-period-like plasticity for social and reward learning (44). Consistent with this, preliminary neuroimaging and neurotrophic-marker changes have accompanied clinical improvement after ketamine in adolescents specifically (28).

Multiple routes of administration are used by practitioners treating depression and other psychiatric conditions, each with its own unique pharmacokinetics, including intravenous, intramuscular, intranasal, sublingual, subcutaneous, epidural, anal, and oral delivery. There are basic axioms for ketamine administration: The route of administration determines the amount of ketamine absorbed, and the time to onset of ketamine’s effects. Ketamine’s effects relate to the total amount of ketamine absorbed and the sensitivity of the recipient to ketamine. Sensitivity is a far more impactful factor than the number of milligrams per kilogram (17).

### Safety of ketamine

While the neurotoxicity of ketamine has been demonstrated in animal models, its clinical relevance is questionable. Examples of studies showing both neurodegeneration and neuroprotection in the developmental period are cited (45–47). Relevant factors include 1.) susceptible developmental age, 2.) high dose of the anesthetic, and 3.) long duration of exposure. When neurotoxicity in animals is demonstrated, doses are much higher and for a much longer duration than in clinical use. A clinical manifestation or phenotype of anesthetic-induced neurodegeneration has not been identified in humans, providing reassurance for the continued use of general anesthesia in children (48).

There are numerous clinical studies of ketamine for anesthesia and procedural sedation in children and adolescents indicating its safety (46, 49–51). In addition, a study of nine children inadvertently given 5–100 times the intended dose of ketamine in the emergency department showed no adverse outcomes, although prolonged sedation occurred in all cases and four experienced brief respiratory depression (52).

The critical time period for neurocognitive damage in primates and humans, appears to be from *in utero* to 3 years, with the possibility of more time due to ongoing synaptic plasticity. There is controversy and ambiguity with respect to this with both ketamine and GABA antagonist anesthetics. The byword is caution in neonates and young children. Animal studies contribute to this complexity with evidence of both neurotoxic and neuroprotective impacts from ketamine (53).

Long-term pediatric use of ketamine on a repeated basis has largely been confined to analgesia with the demonstration of its safety. In adults, long-term continuous infusion for pain has been reviewed with three studies of 4–14 days duration without significant side effects and at doses higher than our episodic intermittent use (54). There is a clear need for prospective studies of long-term effects.

Given ketamine’s episodic, intermittent, and low-dose usage for psychiatric treatment, based on our experience we propose that the effects of continuous mood stabilizers, antipsychotics, stimulants, tranquilizers, and antidepressants present a much greater and unassessed risk to adolescent brain maturation. Furthermore, the negative effects of these on emotion and cognition are well described. The risks to the youth of emotional disorders and morbidity, including suicide, self-harm, eating disorders, impulsivity, alcohol, and drug abuse, outweigh the incompletely described potential risks of ketamine use in a controlled clinical application.

### Ketamine and ketamine-assisted psychotherapy

We provide a comprehensive therapy experience that begins with getting to know each patient and their families and pays full attention to the subjective experience of the ketamine and integrating that experience into their sense of identity, their embodiment, and their relationships (17). The subjective experience of ketamine, or Signature, can be characterized as a temporary respite from the ordinary mind and its habitual obsessions and preoccupations, which facilitates an increased capacity to observe and let go of dysregulated thoughts and behavior patterns. Psychotherapy provides support for a full expression of the subjective experience of ketamine within a conducive non-medicalized setting (16, 17, 35, 55). Our work with adolescents is always in the context of family therapy, including parents and situating adolescents in their homes, schools, and social matrixes. The therapeutic presence is continuous throughout the 3-hour session and begins with Preparation, followed by the ketamine experience, followed by Integration. Subsequent Integration sessions follow the ketamine session as follow-up and as appropriate to the clinical process.

In this relatively early stage of KAP administration with adolescents, families seek our ketamine program after failure with conventional treatment that may include multiple antidepressants, antipsychotics, and mood stabilizing agents, and for a wide variety of clinical presentations. Parents come to us with feelings of confusion, frustration, helplessness, and conflict exacerbated by fear for their child and their own sense of inadequacy. Often, they are in eating disorder programs and have had multiple therapists. Diagnosis in adolescents often consists of multiple labels. It is our view that in general, these diagnoses describe surface symptoms and behaviors rather than the underlying psychodynamic picture. Rather, we most often find trauma, and dysfunctional family situations to be at base with diagnostic flux with the course of treatment. We refer to our previous report (1) for more details.

### Treatment protocol

Written informed consent is reviewed with parents and adolescents and signed by all involved after satisfying any concerns and questions. The possibility of withdrawal from treatment is present at any time, before, or during treatment, up to the period of administration of the ketamine. Our informed consent is detailed as to effects and potential adverse effects and the rationale for ketamine’s use. This is repeated in verbal interaction.

Once appropriateness for treatment has been established, we administer ketamine using two routes of administration: sublingual (SL) or intramuscular (IM) injection. These routes of administration can be administered safely in an office practice where the environment facilitates a comfortable setting for the ketamine experience, in contrast to the often medicalized setting of IV use. Multiple dosing strategies are available for each route of administration and are tailored to patient needs and responses. Our dosage ranges are: for the sublingual route 100-300mg rapid dissolving tablets, the saliva held in the oral cavity for 15 minutes and then expectorated; and for the Intramuscular route 25mg-100mg per session depending on the therapeutic objectives.

The route of administration (SL or IM) is clinically determined and is provided in a carefully tailored manner beginning with a low first dose and stopping at a dose that is effective for ego-dissolution. IM dosing allows for flexibility of the clinical choice of depth of experience: from mild alteration of consciousness to full ego-dissolution and an internalized experience separated from external sensory input. This state is valued for ketamine’s effects for a variety of reasons. Primarily, it is a time-out from the symptomatic and obsessional preoccupations that are the expression of the patient’s suffering. In the case of an adolescent, it may be felt as a relief from the pressures causing symptomatic behavior, allowing for a reformation of consciousness and attitudes. With therapeutic guidance, this experience opens new possibilities for self-understanding, self-control, and behavior. The achievement of this generally requires repetition of ketamine experiences, bonding with therapists, and understanding and support for the psychosocial culture and its participants. In addition, the ketamine experience itself confirms the adolescent’s inherent capacities and imagination, which have become blocked by the parts that are dysregulated and reactive. The ketamine space is interesting, free of usual concerns, and tends to have a neutral to loving and self-appreciating effect. This positive reclamation may well lead to a reduction in self-loathing, despair, suicidality, and reactivity, opening the door to healing.

The administration of SL lozenges at home is a mainstay of our KAP work. We are aware that at-home ketamine lozenge use has become a major commercial enterprise and is open to critical scrutiny in terms of the thoroughness of the programs that distribute the lozenges, controls on use and dosage, and a lack of psychiatric support (56, 57). Our clinical provision of ketamine for at-home sessions is quite different in being far more supervised and embedded in our face-to-face ongoing KAP program. For our adolescents, this means that parents are in possession of a limited quantity of the lozenges we prescribe, with no opportunity for automatic refilling. Adolescents must request lozenges from parents. A parent, or both parents, is always present for at-home sessions, and psychotherapy is incorporated through integration sessions that follow each at-home session. Parents are supervising sessions under the direction of our practitioners. We are available for consultation and the handling of potential crises or safety issues (which have not occurred to date, with at-home use under regular review). At-home use increases session frequency, reduces cost, and expands access, all of which contribute to treatment success. With careful monitoring of prescriptions and supervising timeframes for use, we have had no safety issues, misuse, or diversion (1, 16, 17). At-home sessions are supervised online and with Integration sessions as an integral part of the therapeutic process.

Both psychodynamic and Internal Family Systems approaches were the most frequently employed methodologies. The family-therapy approach varied depending on the composition of the family, the members in attendance, and the particular situation of the family. Parents were counseled as couples and separately; as many of the families were divorced or separated, parents or entire family systems were not always in attendance together.

We refer to Wolfson et al. (1) for more details on clinical practices in this population.

## Methods

### Design, setting, and ethical oversight

We conducted a retrospective chart review of consecutive adolescents and young adults who received ketamine-assisted psychotherapy (KAP) at the Center for Transformational Psychotherapy in San Anselmo between February 2020 and August 2024. The clinic is a community-based outpatient service that delivers both preparation-and-integration psychotherapy with ketamine administration in a single facility designed for comfort and reflection.

Written informed consent was obtained from each patient and at least one parent or legal guardian. The consent document explains the pharmacology of ketamine, potential benefits, transient adverse effects (e.g., nausea, dissociation, elevations in blood pressure), and the risk of psychological discomfort. Families were reminded that participation is voluntary and that they may withdraw at any time before administration of ketamine without penalty.

Confidentiality is addressed explicitly and is essential to working successfully with adolescents. What constitutes a therapist’s obligation to break confidence is made clear from the outset to both children and parents. The creation of trust and respect for adolescent autonomy is of primary concern throughout the therapeutic process.

Charting was both manual and electronic, managed through *RedCap* (Research Electronic Data Capture, Vanderbilt University Medical Center), a HIPAA-compliant data platform.

### Participants and eligibility

The clinical group was defined by a pre-specified filter applied to the full clinic database rather than by a hand-curated list. Of the clinic’s REDCap records, 61 were adolescents and young adults (ages 14–24) who initiated at least one KAP session; 38 of these began treatment within the study window (February 2020–August 2024); 30 completed at least three dated clinical visits; 23 had at least two BDI or HAM-A assessment timepoints, so that a within-patient change could be computed; 20 remained after excluding three patients flagged at chart review as clinical outliers (primary psychotic or bipolar/manic presentation); and 19 remained after excluding one otherwise-eligible patient for whom no chart-review record was available for clinical verification (mean age ± SD 19.3 ± 3.1 years; see **Table 1**). The 19 in-window patients who were excluded were somewhat more symptomatic at intake than those retained (mean BDI 35.2 vs 31.5; HAM-A 26.7 vs 20.3) and completed fewer clinical visits (6.7 vs 9.4); the analyzed clinical group is therefore enriched for patients who engaged with and tolerated treatment, and observed improvements may not generalize to the most severely affected or earliest-discontinuing patients.

**Table 1:** Demographic and intake measure summaries.

Demographics						
sex	N	Mean age	Median age	Min age	Max age	sd
female	11	18.4	18.6	14	23.9	3.0
male	7	20.2	19.3	16	23.7	3.1
non-binary	1	23	23	23	23	NA

Intake easures						
	N	mean	median	min	max	sd
BDI	19	31.5	32	11	50	12.4
HAM	19	20.3	19	6	35	8.9
Resilience	12	11.00	10	9	14	1.60
ACE	10	0.70	0	0.00	4	1.34
PCL-5	13	38.85	39	6.00	70	17.93

### Baseline and outcome measures

The primary clinical outcomes were the longitudinal trajectories of Beck Depression Inventory–II (BDI-II) and Hamilton Anxiety Rating Scale (HAM-A) scores. Secondary exploratory outcomes included the 30-item Mystical Experience Questionnaire (MEQ-30), the Eating Disorder Inventory (EDI), and practitioner-recorded psychedelic effects reported by the subject. Other surveys used to characterize the clinical group included the PTSD Checklist for DSM-5 (PCL-5), the Adverse Childhood Experiences questionnaire (ACE), and a Resilience survey. Overall change in score was defined as the final recorded value minus the first. Negative change indicates improvement for BDI and HAM scores, whereas positive change reflects greater psychedelic experiences derived from the MEQ and EDI.

Diagnosis at baseline was established through a multi-source procedure that integrates the clinic’s structured intake questionnaire, review of external medical records, and information from parents or outside therapists. For analytic clarity, individual DSM-5 diagnoses were collapsed into nine disorder families: post-traumatic stress, attention-deficit/hyperactivity, depressive, eating, anxiety, neurological, substance use, obsessive–compulsive, and other conditions.

### Statistical analysis

Descriptive statistics and visualizations were used to summarize demographics, diagnostics, psychedelic experiences, and outcome measures. To test whether overall clinical group BDI and HAM scores changed over time, we fitted random-intercept linear mixed effects models with visit number as a fixed effect and patient level random effects with the lme4, lmerTest, merTools packages (58–60).

As a sensitivity analysis for the shape of the trajectories, we additionally fit penalized generalized additive models (a smooth function of visit number with patient-level random intercepts) using the *mgcv* package (61).

To quantify the magnitude of individual changes in measure (from intake to final recording) for each subject, we calculated Cohen’s d as an effect size index for paired designs (62–64). Taking BDI as an example measure, we calculate the effect size as the standardized change for each subject, $d_{BDI,i}=\;\Delta BDI_{i\;}/\;SD,\;i=1,\ldots ,n,$ where SD is a reference standard deviation calculated using the pre-treatment scores. A negative Cohen’s *d* statistic for BDI or HAM scores indicates improvement, whereas a positive value reflects a worsened condition. Effect sizes were interpreted using benchmark ranges of: Large (|d| > 0.8), Medium (0.5 < |d| ≤ 0.8), Small (0.2 < |d| ≤ 0.5), and Negligible (|d| ≤ 0.2) (65). To examine whether intake symptom severity differed between patients who achieved medium or large treatment responses and those who did not, we used two-sample Wilcoxon rank-sum tests comparing first-recorded BDI and HAM scores across response groups. A normal approximation was used for p-value calculation given the presence of ties.

Pearson correlations were used to examine the association between overall change in scores and total number of KAP sessions. Because of the modest sample size and exploratory scope of this study, we emphasize that inferential significance based on p-values should be interpreted as descriptive rather than confirmatory. Analyses were performed in R (66). We additionally examined whether the availability of BDI and HAM-A scores was related to visit number, baseline symptom severity, or total treatment length, to assess whether intermittent recording could bias the estimated trajectories.

Not all intake instruments were administered uniformly across the study period; the Resilience scale (n = 12/19), ACE questionnaire (n = 10/19), and PCL-5 (n = 13/19) were incorporated into the intake protocol at different points during the clinic’s operation and are therefore not available for the full clinical group. In contrast, BDI and HAM were recorded intermittently throughout treatment for all 19 patients.

## Results

### Clinical group profile

The clinical group of adolescents consisted of 11 females, 7 males, and one non-binary individual. The typical subject was in their late teens (mean = 19.3, SD = 3.1), and the average age of male subjects was higher than that of females (20.2 vs. 18.4) with similar ranges (**Table 1**, **Figure 1A**). All subjects had at least 3 clinical treatment visits. The average duration of treatment was 9.7 visits (SD = 8.4); the majority of patients attended 10 or fewer visits (15/19), with 4 patients attending for more than 15 (**Figure 1B**). Visits were conducted at individualized intervals, approximately monthly on average (median 28 days between consecutive visits). Additionally, a majority of patients (17/19, 89%) received at least one home ketamine session in addition to clinic visits.

**Figure 1:**
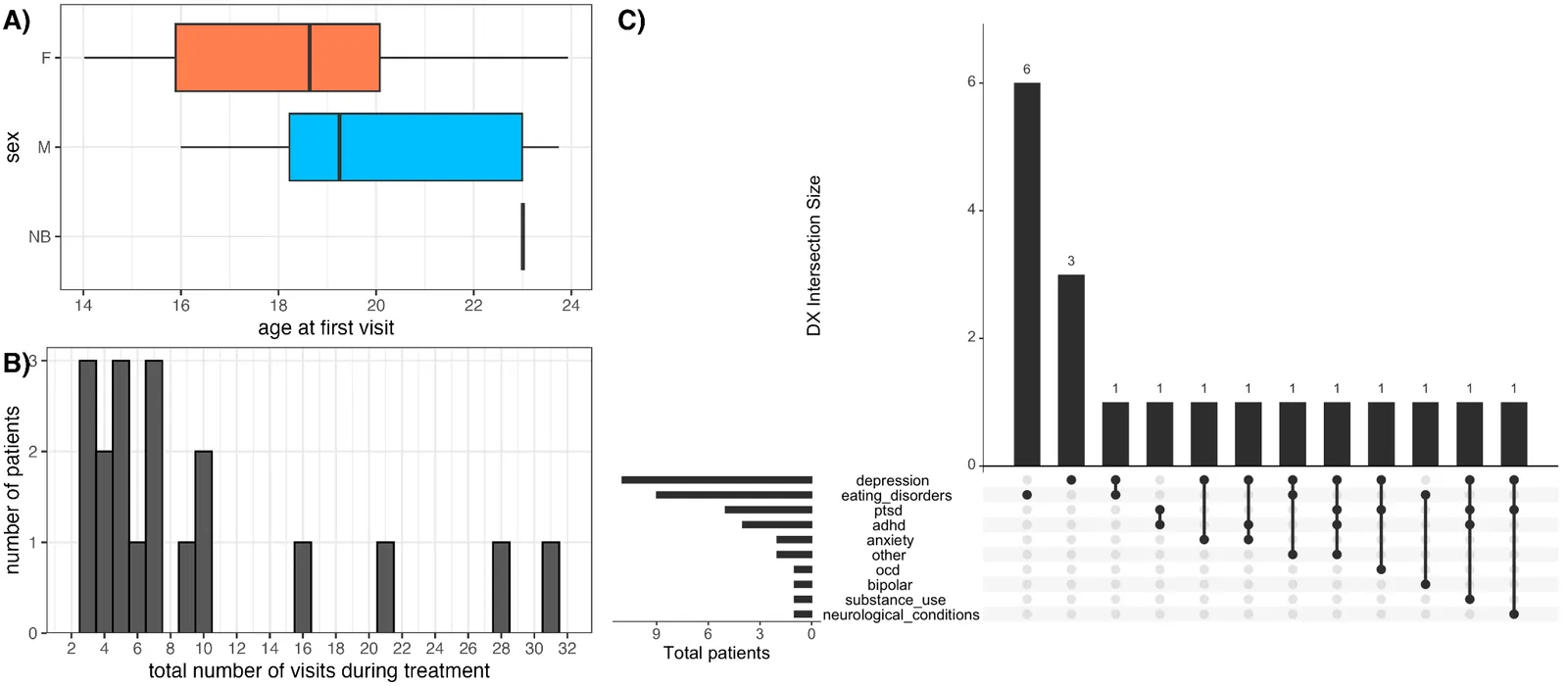
**(A)** Age distribution by sex, **(B)** Distribution of number of clinical visits during treatment, **(C)** Distribution of the 9 diagnostic families throughout treatment. The “Other” category consists of the following diagnoses: delusional disorder and unspecified stress reaction. Note that diagnostic burden is substantial: nearly half of patients carried diagnoses spanning two or more disorder families.

The clinical group exhibited a high level of diagnostic heterogeneity: half (10/19, 52.6%) of patients held diagnoses from 2 or more diagnostic families (range: 1–4 families; mean = 1.95 diagnostic families per patient). Depression, eating disorders, PTSD, and ADHD comprised the most common diagnostic families represented in the clinical group (**Figure 1C**).

**Table 2** depicts a pre-/post-treatment snapshot of overall benefits received, risk factors reduced, and adverse effects experienced by the clinical group. Physician and patient reports indicate that 68.4% of patients experienced an overall benefit from treatment (13/19; **Table 2**). Specifically, treatment reduced the severity of suicidality at very high rates in at-risk patients; eating-disorder outcomes were inconsistently recorded and are reported descriptively. Treatment also resulted in a reduction of the average number of medications a patient needed by about 0.37 prescriptions per patient. No hospitalizations were experienced during the treatment interval.

**Table 2:** Risk factors and benefit of treatment rates for the clinical group.

Meds				
n	Sex	Avg no. meds at intake	Avg. no of meds at termination	Avg. reduction of meds
11	F	1.36	1	0.33
7	M	2.29	1.71	0.57
1	NB	2	3	-1

Suicidality				
	Sex	Suicidality risk present at intake	Suicidality risk reduced	
	F	6/11 (54.5%)	6/6 (100%)	
	M	6/7 (77.7%)	6/6 (100%)	
	NB	1/1 (100%)	1/1 (100%)	

Hospitalization				
	Sex	Avg. # of Hospitalizations at Intake	Fraction of Indivs. with Hospitalization History at Intake	New Hospitalizations during Treatment
	F	0.455	2/11 (18.2%)	0
	M	0.429	2/7 (28.6%)	0
	NB	0	0/1 (0%)	0

Eating disorders (ED)				
	Sex	ED history at intake	ED reduced	
	F	7/11 (63.6%)	3/7 (42.9%)	
	M	0/7 (0%)	NA	
	NB	1/1 (100%)	1/1 (100%)	

Overall benefit from treatment (physician notes)				
	Sex	Yes	Partial	No
	F	8/11 (72.7%)	1/11 (9.1%)	2/11 (18.2%)
	M	4/7 (57.1%)	2/7 (28.6%)	1/7 (14.3%)
	NB	1/1 (100%)	0/1 (0%)	0/1 (0%)

Denominators for suicidality/ED reduced columns reflect only those patients who presented the risk at intake.

### Psychometric trajectories

Most individuals demonstrated a downward (improving) trend in BDI and HAM scores with moderate fluctuations (**Figure 2**). A handful of patients with a low number of sessions plateaued before stopping treatment altogether. Linear mixed effect modeling of longitudinal trajectories of BDI and HAM scores revealed that at the group-level, more visits are associated with greater reduction in depression and anxiety indices (**Table 3**). BDI scores are expected to decrease on average by roughly 0.649 points per visit (95% CI: [−0.897, −0.402]). HAM scores are expected to decrease on average by 0.213 points per visit (95% CI: [−0.441, +0.014]), a trend that did not reach statistical significance (p = 0.069).

**Figure 2:**
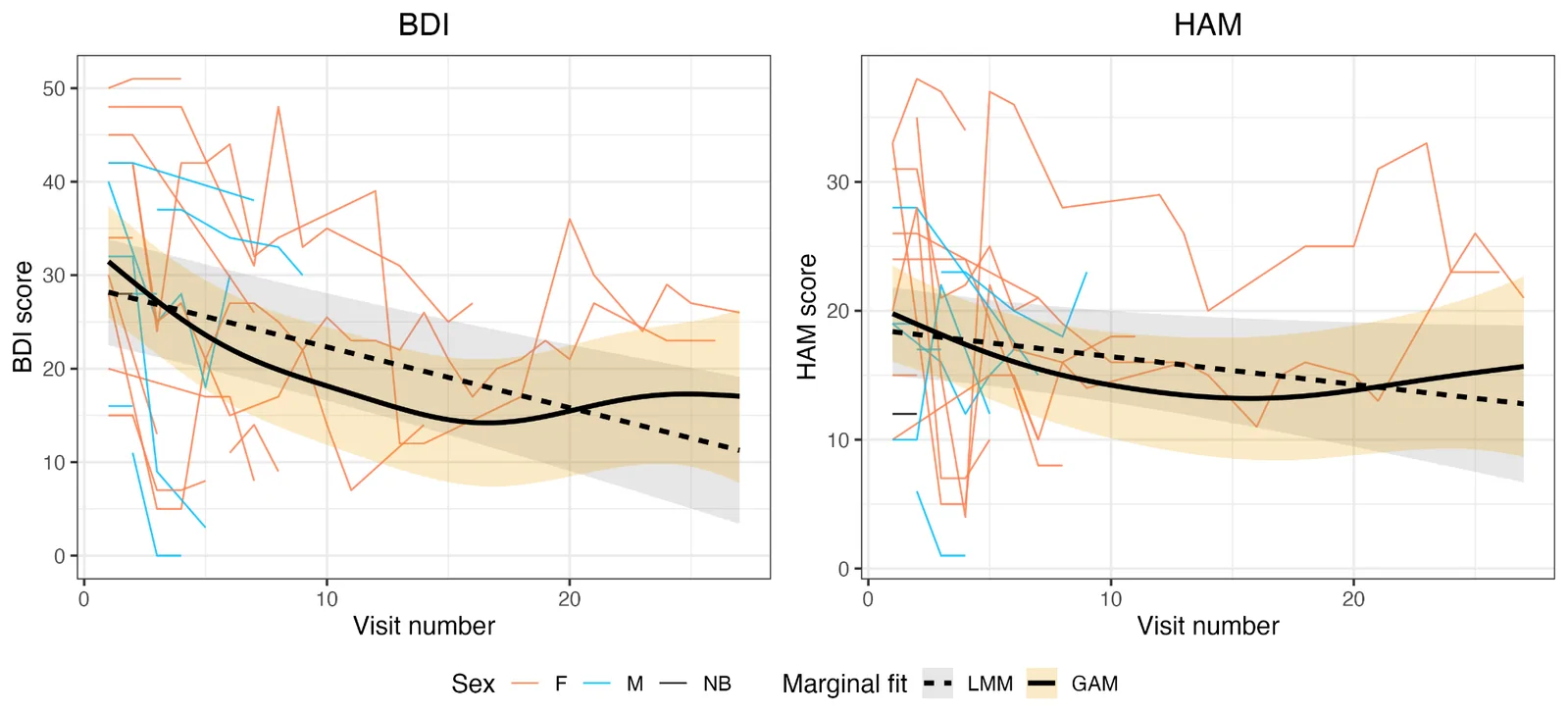
Longitudinal trajectories of BDI (left) and HAM (right) scores against visit number. Thin lines are individual patients (red, female; blue, male; black, non-binary). Overlaid are two fits: the linear mixed-model (dashed) and the GAM (solid), each with a 95% confidence band. Although the group-average trend is downward, the few patients with the longest courses show flat or rising scores at their final visits, and the GAM’s modest late rebound is estimated from these patients.

**Table 3:** Summary of fixed effects for linear mixed models predicting BDI and HAM scores using visit number.

BDI response	Coefficient	SE	t	p-value
Intercept	28.8267	2.8435	10.138	~0
Visit #	-0.6495	0.1265	-5.135	~0

HAM response	Coefficient	SE	t	p
Intercept	18.5990	1.7469	10.647	~0
Visit #	-0.2132	0.1160	-1.838	0.069

These coefficients suggest that across the average across the average 9.7-visit course of treatment, the average BDI score dropped approximately 6.3 points while the average HAM score dropped by roughly 2.1 points.

Because several patients with longer treatment courses showed a slight upturn in scores at their final visits, we assessed whether a single linear trend adequately summarized the trajectories. The generalized additive model provides a non-linear fit over the linear model for both measures. The estimated GAM fits describe front-loaded improvement, steeper than the linear fit over roughly the first ten to fifteen visits, before experiencing a modest rebound among the small number of longest-treated patients. This rebound is estimated with wide confidence bands and rests on few patients; we therefore retain the linear model as our primary summary of the average effect, while noting that the bulk of measurable improvement accrues early in treatment and then plateaus.

### Therapeutic treatment response signals and effect sizes

Focusing on overall change in psychometric measures, we observed a significant negative correlation between the number of visits during treatment and ΔBDI (r = −0.54, 95% CI [−0.80, −0.11], p = 0.018) and ΔHAM (r = −0.58, 95% CI [−0.82, −0.18], p = 0.009; **Figure 3**, **Table 4**). Using Cohen’s *d* to quantify the effect sizes of individuals’ changes in measures, we observed that approximately 63.2% (12/19) of patients experienced a medium or large effect in the reduction of their BDI score and roughly 47.4% (9/19) of patients experienced a medium or large effect in the reduction of their HAM score (**Figure 4**).

**Figure 3:**
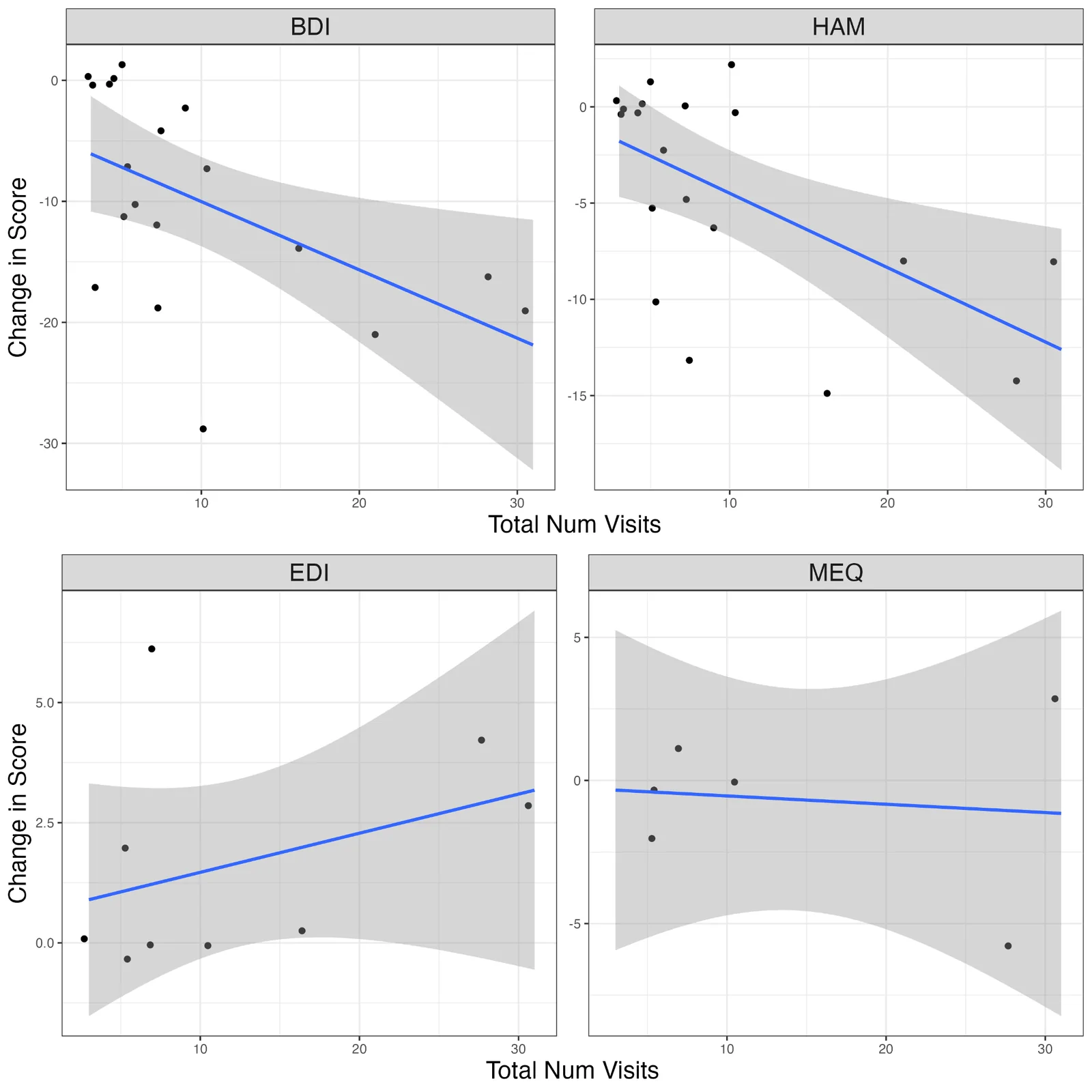
Change in scores vs. total number of visits. Points are jittered slightly to avoid overplotting.

**Table 4:** Correlation between change in measures and total number of visits.

Measures	n	Correlation	SE	t	p-value
BDI	19	-0.537	0.205	-2.63	**0.0018**
HAM	10	-0.584	0.197	-2.97	**0.0090**
EDI	9	0.378	0.350	1.08	0.3200
MEQ	6	-0.113	0.497	-0.23	0.8300

EDI and MEQ results are exploratory and should be viewed with caution because of low sample sizes (n = 9 and n = 6, respectively). Bold values indicate statistical significance at the \alpha = 0.05 level.

**Figure 4:**
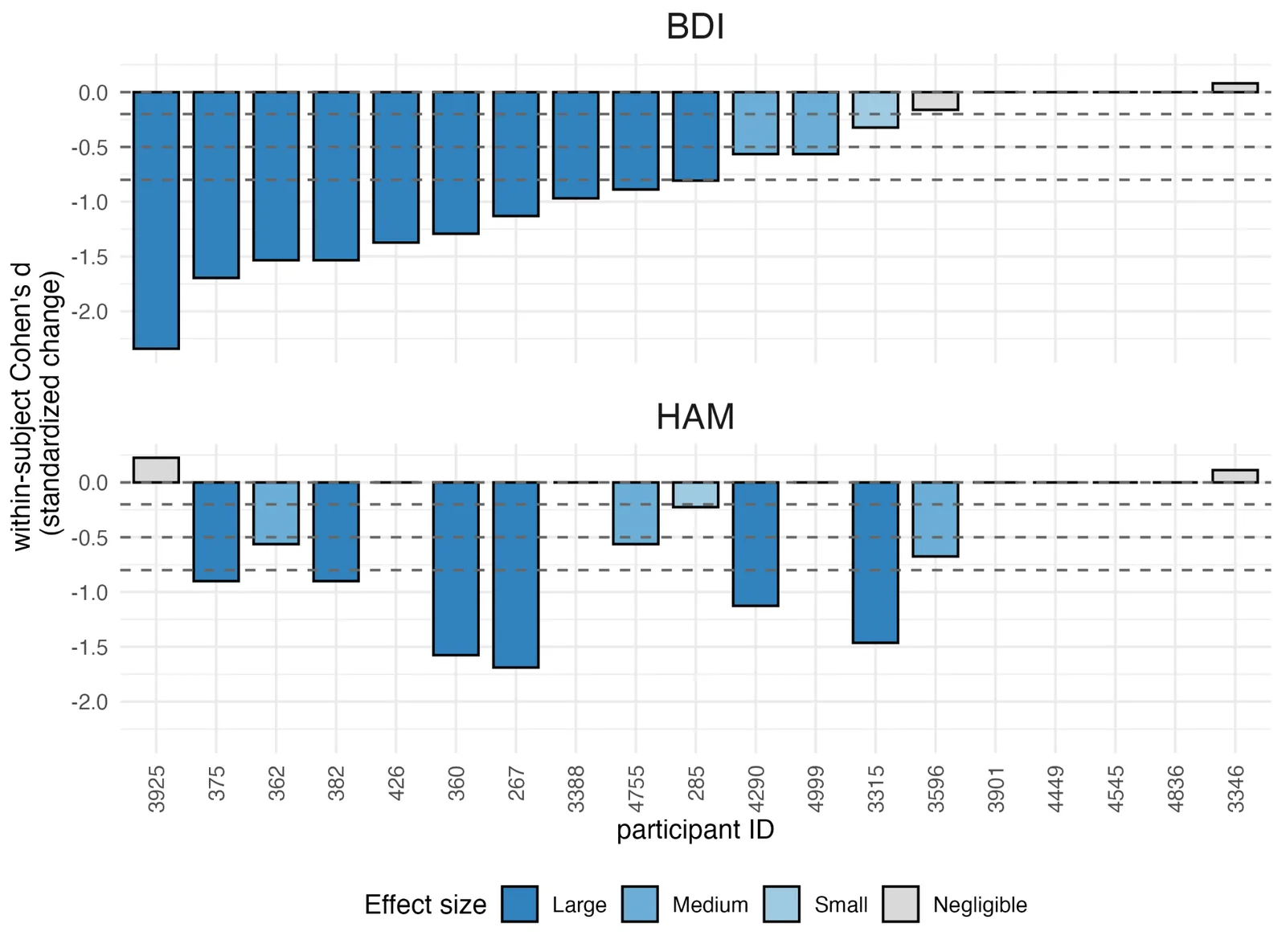
Within-subject effect sizes (Cohen’s d) for BDI (top) and HAM (bottom) across all 21 patients. Patients are sorted by ascending order of BDI effect size (largest improvement leftmost). Bar color indicates effect size magnitudes using the following ranges: Large (|d| > 0.8), Medium (0.5 < |d| ≤ 0.8), Small (0.2 < |d| ≤ 0.5), and Negligible (|d| ≤ 0.2).

BDI and HAM were recorded intermittently rather than at every visit (present at approximately 59% and 54% of visit-rows, respectively), so change scores reflect the first and last available observation per patient. Score availability declined modestly at later visits (logistic regression of observed-versus-missing on visit number: BDI: p = 0.03; HAM: p = 0.006), so the estimated trajectory beyond roughly the fifteenth visit rests on very few observations, consistent with the wide confidence bands there. Coverage was, however, essentially unrelated to baseline depression severity (BDI: r = −0.02) and only weakly related for anxiety (HAM: r = +0.18), arguing against assessment being triggered by clinical state. We therefore expect little bias in the densely sampled early-treatment period and interpret the sparse late-visit tails with caution.

Patients with small or negligible HAM reductions entered treatment with lower anxiety burden (mean intake HAM = 16.9, SD = 7.2) than medium or large responders (mean intake HAM = 24.1, SD = 9.4), though this difference did not reach statistical significance (Wilcoxon W = 22.5, p = 0.072); their scores were also virtually unchanged by discharge (mean last HAM = 17.0 vs. 14.8 in responders), consistent with a floor effect limiting measurable improvement. The same directional pattern was weaker and non-significant for BDI, where patients with low or negligible reductions entered with only slightly lower depressive symptom burden (mean intake BDI = 29.9, SD = 13.7) than medium or large responders (mean = 32.5, SD = 12.1; Wilcoxon W = 36.5, p = 0.671). These findings are suggestive of, but do not establish, greater measurable benefit among patients with more severe baseline symptoms, a hypothesis worth examining in adequately powered future studies.

Neither secondary subjective-experience measure showed a reliable association with treatment exposure: the correlation between number of sessions and change in EDI was small and non-significant (r = 0.38, 95% CI [−0.38, 0.83], n = 9), and the MEQ correlation was effectively null (r = −0.11, 95% CI [−0.85, 0.77], n = 6). Given the very small samples, these measures are reported as exploratory only, and we draw no conclusion about whether ego-dissolution or mystical experience accrues with continued treatment.

We additionally examined whether the magnitude of the psychedelic experience was related to symptom change. Among the six patients with MEQ data, higher average MEQ scores were associated with larger reductions in depression, though not significantly (r = −0.58, 95% CI [−0.95, 0.44], p = 0.23, n = 6), with a weaker association for anxiety (r = −0.27, p = 0.60). Given the very small sample, we regard this as an exploratory, descriptive finding.

### At-home therapy

A majority of patients (17/19, 89%) received at least one home ketamine session in addition to clinic visits. Patients who received home sessions showed greater average improvement in BDI than the two patients who did not (Wilcoxon W = 4, one-tailed p = 0.048), but this comparison should not be interpreted as an established home-session effect. Both patients without home sessions contributed only a single BDI observation, so their change scores are zero by construction rather than by a measured absence of improvement, and both also completed far fewer office visits; home-session exposure therefore cannot be disentangled from overall treatment dose. We report the at-home modality as a feasible and widely used component of care rather than as an independent driver of outcome.

### Adverse effects

Ketamine was generally well tolerated across the 167 documented sessions. Nausea was the most common adverse event, affecting 6 of 19 patients (31.6%) and occurring in 9 of 167 sessions (5.4%). Of the 6 affected patients, 4 were female and 2 were male. Vomiting was rare, occurring in 1 patient across 1 session (0.6%). No agitation, hypertension, headache, or insomnia was documented. Notably, 9 of 19 patients received antiemetic prophylaxis or treatment (oral ondansetron or occasionally scopolamine patches) at some point during their care. No new hospitalizations occurred during the treatment interval (**Table 5**).

**Table 5:** Documentation of adverse effects. 4 females and 2 males had nausea.

	Nausea	Vomiting	Agitation	Hypertension	Headache	Insomnia
Patients affected	6/19 (31.6%)	1/19 (5.3%)	0/19 (0%)	0/19 (0%)	0/19 (0%)	0/19 (0%)
Sessions affected	9/167 (5.4%)	1/167 (0.6%)	NA	NA	NA	NA

9 of the 19 patients received Zofran or Scopolamine at some point during treatment.

## Discussion

KAP delivered in a family-centered outpatient setting is feasible and well tolerated in diagnostically complex adolescents and young adults, a population for whom conventional treatment has frequently proved inadequate. Our data provide evidence that continued engagement with the program is associated with clinically meaningful reductions in depression and anxiety, indicators of response that are apparent across both individual effect sizes and group-level mixed-model estimates. Because treatment was uncontrolled and patients self-selected into longer courses, we describe this as an association between treatment exposure and symptom reduction rather than a dose-response effect. Adverse effects were mild and transient, consistent with experience in adults. Nausea was the only notable adverse event and occurred in fewer than 6% of individual sessions, largely manageable with standard antiemetics. The absence of agitation, cardiovascular events, or hospitalizations across 167 documented sessions supports the feasibility of outpatient KAP delivery in this age group.

A larger number of sessions was linked to larger reductions in depression, anxiety, and other symptoms. Reduction in severity, decrease in conventional medicines, and graduation from management programs such as those for eating disorders were also evident. The number of sessions administered reflects the degree of acuity from the onset of our patient contact, the time for development of trust with therapists, the process and success of concomitant family therapy, and the development of autonomy and diminishing self-harm. The KAP therapeutic process is a complex undertaking and in future work, we will add to the description of our methodology and pursue validation of the severity reduction results presented here.

Dissociative/psychedelic experiences are an essential part of the ketamine experience (17). In this patient population, they were well tolerated, accepted, and adopted as an important aspect of treatment. A gradual approach to the introduction of ketamine is fundamental to our treatment, leading to an assessment of individual sensitivity to the medicine, strategies for its therapeutic use, and building a sense of acceptance and safety by our patients and their families.

Based on our experience as practitioners of KAP for adolescents, young adults, and their families, we encounter and are aware of parental concerns and fears surrounding ketamine administration to their children. Most of the parents who sought our care framed it as a “last resort” when all else had failed, or when treatment was having serious impact on lives in terms of time, numbers of practitioners, family stress and resultant dysfunctionality, hopelessness, fear for safety, ongoing self-harm, and suicidality. Clinically meaningful symptom reduction was observed in this context without serious adverse events. Families who completed three or more sessions consistently reported diminution in the severity of the presenting problems, including suicidality, self-harm, and family dysfunction, though we acknowledge that not all of these outcomes are fully captured in our quantitative measures.

Family therapy was provided differentially to the various family formations we encountered: intact partnerships, broken and divorced partnerships with cooperating, or struggling parents, single parents, and reformed new partnership constellations. Therapists practiced within their various trainings adapted to ketamine’s formative impact on treatment. Methodologies included psychodynamic, Internal Family Systems (IFS), Gestalt, and Systemic Family Therapy with flexible practices adjusted to the situations encountered and the arc of treatment.

A frequently expressed concern regarding ketamine in adolescents is its potential impact on the developing brain. Our clinical observations run counter to this concern: over the course of treatment, and by clinical impression rather than formal testing, symptom burden decreased while educational and social functioning, autonomy, and self-direction appeared to improve, and no patient showed signs of cognitive decline, substance misuse, dependence, or diversion. These are chart-review observations rather than instrumented outcomes: no standardized neurocognitive battery or functional-status measure was administered (the clinic’s Global Assessment of Functioning field was not scored), and we list this among the study’s limitations. A part of our approach has been to provide an openness to discussion of self and peer use of substances. Responsible use in our clinical setting has functioned as education and a model for what adolescents encounter in their lives and peer groups.

There are several limitations to the depth of our review. We present a small, uncontrolled sample with a varied degree of follow-up and some dropouts. Survey measures were often intermittently recorded, and changes in scores reflect the first and last available observation per patient rather than a fixed assessment window. Quantification of the effects of family therapy is not provided. Several qualitative claims regarding functional improvement and substance non-misuse rely on practitioner and patient reporting rather than validated instruments.

Our safety observations are limited to the active-treatment window (median 5.6 months); we did not conduct systematic surveillance after the final session, so the durability of benefit and the possibility of delayed harms remain uncharacterized. These limitations point to the need for pre-registered prospective studies with larger, controlled samples, standardized collection of BDI, HAM, EDI and MEQ surveys at every session, validated measurement of family therapy outcomes, and longer follow-up to characterize the durability of observed improvements.

## Conclusions

Our longitudinal analyses suggest that continued and consistent KAP sessions may yield improvements in mood, anxiety, and subjective positive-state measures for adolescents, young adults, and their families. Because the design is uncontrolled and the sample is small, these results are hypothesis-generating rather than confirmatory: they establish feasibility and motivate but cannot by themselves establish the efficacy of office-based KAP in this population. Within these limits, the treatment-resistant nature of our clinical group and the absence of serious adverse events across 167 sessions are encouraging, and our findings corroborate the emerging view that ketamine’s therapeutic effects may be enhanced in a psychotherapy context. We regard KAP embedded in an intensive psychotherapeutic program as a promising candidate for adolescents and young adults for whom conventional care has proved insufficient, and one that now warrants evaluation in adequately powered, controlled trials.

Further, our findings support the emerging view that ketamine’s therapeutic effects are meaningfully enhanced in a psychotherapy context. Our work with this small clinical population is evidence that ketamine embedded in an intensive psychotherapeutic program represents a positive, safe, and much needed additional tool for the treatment of a youth population facing an escalating public health crisis. Larger, controlled studies are needed to further validate these results.

## Data Availability

The datasets presented in this article are not readily available because the datasets in this study were derived from electronic health records of patients, which contain protected health information and thus cannot be shared without appropriate data use agreements. Requests to access the data can be directed to the corresponding author.
